# CD3^+^ Macrophages Deliver Proinflammatory Cytokines by a CD3- and Transmembrane TNF-Dependent Pathway and Are Increased at the BCG-Infection Site

**DOI:** 10.3389/fimmu.2019.02550

**Published:** 2019-11-07

**Authors:** Adriana Rodriguez-Cruz, Dominique Vesin, Lucero Ramon-Luing, Joaquin Zuñiga, Valérie F. J. Quesniaux, Bernhard Ryffel, Ricardo Lascurain, Irene Garcia, Leslie Chávez-Galán

**Affiliations:** ^1^Department of Biochemistry, Faculty of Medicine, Universidad Nacional Autónoma de México, Mexico City, Mexico; ^2^Department of Pathology and Immunology, Faculty of Medicine, Centre Medical Universitaire, University of Geneva, Geneva, Switzerland; ^3^Laboratory of Integrative Immunology, Instituto Nacional de Enfermedades Respiratorias “Ismael Cosío Villegas”, Mexico City, Mexico; ^4^Laboratory of Immunobiology and Genetics, Instituto Nacional de Enfermedades Respiratorias “Ismael Cosío Villegas”, Mexico City, Mexico; ^5^Experimental Molecular Immunology and Neurogenetics (UMR7355), CNRS and University of Orléans, Orléans, France; ^6^Hospital Nacional Homeopático, Secretaría de Salud, Mexico City, Mexico

**Keywords:** macrophage, CD3, TNF pathway, pro-inflammatory cytokines, BCG infection

## Abstract

Macrophages are essential cells of the innate immune response against microbial infections, and they have the ability to adapt under both pro- and anti-inflammatory conditions and develop different functions. A growing body of evidence regarding a novel macrophage subpopulation that expresses CD3 has recently emerged. Here, we explain that human circulating monocytes can be differentiated into CD3^+^TCRαβ^+^ and CD3^+^TCRαβ^−^ macrophages. Both cell subpopulations express on their cell surface HLA family molecules, but only the CD3^+^TCRαβ^+^ macrophage subpopulation co-express CD1 family molecules and transmembrane TNF (tmTNF). CD3^+^TCRαβ^+^ macrophages secrete IL-1β, IL-6 IP-10, and MCP-1 by both tmTNF- and CD3-dependent pathways, while CD3^+^TCRαβ^−^ macrophages specifically produce IFN-γ, TNF, and MIP-1β by a CD3-dependent pathway. In this study, we also used a mouse model of BCG-induced pleurisy and demonstrated that CD3^+^ myeloid cells (TCRαβ^+^ and TCRαβ^−^ cells) are increased at the infection sites during the acute phase (2 weeks post-infection). Interestingly, cell increment was mediated by tmTNF, and the soluble form of TNF was dispensable. BCG-infection also induced the expression of TNF receptor 2 on CD3^+^ myeloid cells, which increased after BCG-infection, suggesting that the tmTNF/TNFRs axis plays an important role in the presence or function of these cells in tuberculosis.

## Author summary

In response to physiologic changes or infectious challenge, macrophages play a main role in maintaining cellular homeostasis in tissues. Macrophages have high plasticity, and their differentiation process produces heterogeneous results. Recently, the evidence has increased in regard to a novel macrophage subpopulation called CD3^+^ macrophages that accumulate under both infectious and non-infectious scenarios. Reportedly, TNF plays a role in their generation and presence.

This work provides the phenotypical characterization of two new subpopulations of human CD3^+^ macrophages differentiated from circulating monocytes (MDM). They are CD3^+^TCRαβ^+^ and CD3^+^TCRαβ^−^ MDM; interestingly, they share the expression of classic molecules of macrophages, such as HLA class I, class II and the mannose receptor. Additionally, CD3^+^TCRαβ^+^ MDM co-express CD1 family molecules, transmembrane TNF (tmTNF) and secrete IL-1β, IL-6 IP-10, and MCP-1 by tmTNF- and CD3-dependent pathways. Since CD3^+^TCRαβ^+^ MDM express CD1 molecules, our data suggest that this cell subpopulation can play an important role in pathologies where lipids are abundant. Regarding the CD3^+^TCRαβ^−^ MDM subpopulation, the expression of tmTNF is low, but these cells produce IFN-γ, TNF, and MIP-1β after they are activated by the CD3-dependent pathway. To clarify the *in vivo* role of TNF, we used a mouse model of BCG-induced pleurisy in mouse expressing only tmTNF and not soluble TNF. Our data provided further evidence on the role of tmTNF to regulate the presence of CD3^+^TCRαβ^+^ and CD3^+^TCRαβ^−^ myeloid cells at the infection site.

In summary, we have provided new insights about the characterization and function of these novel macrophage subpopulations which have been related to several conditions, including tuberculosis, malaria, cancer and atherosclerosis.

## Introduction

Macrophages comprise a heterogeneous cell population of myeloid origin and are essential in immune response. Their various functions include the phagocytosis of debris, dead, or infected cells or microbial products and pathogens; the processing and presentation of phagocytosed antigens by major histocompatibility complex class II molecules to activate antigen-specific T cells; the production of pro- and anti-inflammatory cytokines; and tissue repair even under sterile conditions (1). In response to stimuli, macrophages differentiate into two major distinct effector macrophage subpopulations with contrasting functions according to the profile of cytokine delivery (1, 2). M1 and M2 nomenclature has been proposed to refer to macrophages that are activated by IFN-γ and that deliver pro-inflammatory cytokines (M1) vs. those that are activated by IL-4 and deliver anti-inflammatory cytokines (M2) (2).

Tuberculosis (TB) is an infectious disease caused by the *Mycobacterium tuberculosis* (*M. tuberculosis*) bacillus. In 2016, the World Health Organization reported 10.4 million new cases of TB and 1.5 million deaths worldwide (3). During the establishment of mycobacterial infection, an inflammatory response is activated and a large number of leukocytes are recruited from blood vessels to the site of infection, including monocytes, which differentiate into macrophage subpopulations that in turn participate in the formation of granulomas (4). Granulomas have been assigned wide-ranging functions; on one hand, they are proposed as a vehicle to spread the pathogen, and on the other hand, as a successful manner of sequestering the pathogen together with cells producing inflammatory mediators (5).

Tumor necrosis factor (TNF) is a pro-inflammatory cytokine that plays a main role in generating and maintaining the tuberculous granuloma (6). TNF is synthesized as a transmembrane form precursor (tmTNF) and then cleaved by the TNF-α-converting enzyme (TACE) upon activation stimuli to release the soluble form of TNF (solTNF). Both tmTNF and solTNF are bioactive molecules that interact with TNF receptor type 1 and 2 (TNFR1 and TNFR2) to induce cellular activation (7). T cell-derived TNF is required for the formation of granulomas, but to sustain the protective immunity against *M. tuberculosis*, both T cell- and macrophage-derived TNF molecules are necessary (8).

Our previous studies have shown that a *Mycobacterium bovis* bacillus Calmette-Guérin (BCG)-induced pleural infection in TNF KO and double TNFR1 and TNFR2 (TNFR1R2) KO mice was associated with exacerbated inflammation that in turn impaired bacterial clearance and was lethal to the host (9). We also demonstrated that during BCG-induced pleurisy, myeloid-derived suppressor cells (MDSC) accumulated in the pleural cavity and tmTNF expressed by MDSC mediated immune suppression through interaction with CD4^+^ T cells expressing TNFR2. This interaction led to the attenuation of the excessive inflammatory response by the control of the proliferation of T cells producing IFN-γ (10). Those reports together support evidence about TNF playing a main role in controlling mycobacterial infections, both favoring an inflammatory response and activating a regulatory mechanism.

A previous report noted a subpopulation of macrophages expressing the CD3/T cell receptor (TCR)αβ complex (CD3^+^ macrophages), generated by V(D)J recombination, accumulated in granulomas from TB patients. Using an *in vitro* model of BCG infection, the authors confirmed that the infection increased the frequency of CD3^+^ macrophages, and the presence of TNF was essential to maintain this cell subpopulation (11). Recently we demonstrated that BCG infection induces an increase of the recruitment of CD11b^+^CD3^+^ phagocytic cells, and it is dependent of TNFR1 expression on myeloid cells, more interesting, we reported there are two CD3^+^ myeloid subpopulation, one more abundant and phenotypically TCRαβ^−^ and a second TCRαβ^+^ (12).

In this study, we used monocyte-derived macrophages (MDM) from healthy human donors to identify the frequency of CD3^+^ macrophages and to assess the role of TNF and CD3 in the activation and differentiation of CD3^+^ macrophages. Then, we used a murine model of BCG-induced pleural infection to investigate the role of tmTNF vs. solTNF to regulate the presence of CD3^+^ myeloid cells to the infection site. Our data corroborate the presence of human CD3^+^ macrophages, and we also confirmed they are divided into TCRαβ^+^ and TCRαβ^−^ phenotypes. Both macrophage subpopulations deliver a pro-inflammatory cytokine profile but CD3+TCRαβ+ macrophages by both tmTNF- and CD3-dependent pathways, while CD3+TCRαβ- only by a CD3-dependent pathway. We demonstrated that tmTNF expression but not solTNF, is enough to maintain the CD3^+^ myeloid cells within the pleural cavity.

## Materials and Methods

### Human Cells and Animals

The peripheral blood mononuclear cells (PBMCs) were obtained from buffy coats collected in the blood bank at the *Instituto Nacional de Enfermedades Respiratorias Ismael Cos*í*o Villegas*, Mexico City, Mexico. The study was approved by the Institutional Review Board (IRB# B07-16) and was conducted following the principles stipulated in the Helsinki Declaration.

All animal experiments were carried out in accordance with institutional guidelines and approved by the academic ethical committee on animal experimentation and by the cantonal veterinary office from Geneva (CVOG). The protocol assurance number for our CVOG is GE167/14. The CVOG adheres to national guidelines of the ethical principles founded on the CRUS Policy and the Swiss Academy of Medical Sciences (ASSM) and Swiss Academy of Sciences (SCNAT) “Ethical Principles and Guidelines for Animal Research.”

### Preparation of Cells

The peripheral blood mononuclear cells (PBMCs) were isolated from buffy coats by standard Lymphoprep^TM^ (Accurate Chemical-Scientific, Westbury, NY, USA) gradient centrifugation. The monocytes were isolated by positive selection using magnetic microbeads coated with anti-CD14 monoclonal antibody (mAb) in a MACS system (Miltenyi Biotech, Bergisch Gladbach, Germany). Enrichment of the CD14^+^ cell fraction was routinely around 98%, as analyzed by flow cytometry using fluorochrome-labeled anti-human CD14 mAb. The CD14^+^ cells were cultured at 1 × 10^6^ cells/well in 24-well plates (Costar, Ontario, Canada) in RPMI-1640 culture medium (GIBCO, Grand Island, NY, USA), supplemented with 2 mM *L*-glutamine (GIBCO, Grand Island, NY, USA), 100 μg/ml streptomycin, 100 IU/ml penicillin and 10% heat-inactivated fetal bovine serum (GIBCO, Grand Island, NY, USA) for 7 days at 37°C in a humidified atmosphere containing 5% CO_2_. When the cell culture was complete, the viable cells were considered to be monocyte-derived macrophages (MDM) based on the expression of differentiation molecules, as previously reported (13).

### Flow Cytometry

We evaluated cell surface marker expressions on cultured human macrophages using monoclonal antibodies (mAbs) to CD80, CD86, CD11b, CD68, CD14, CD16, HLA-ABC, HLA-DR, CD1a, CD1b, CD1c, CD1d, the TCRβ chain, TCRγδ, CD3ε (epsilon chain), CCR4, CCR7, CXCR1, and TNF. Likewise, to evaluate mouse myeloid cells, we used mAbs to CD11b, CD3ε, TCRβ, TNFR1, and TNFR2. All the mAbs were provided by BioLegend (San Diego, CA, USA). The cells were stained for 30 min, at 4°C in the dark. Then, the cells were fixed by 2% *p*-formaldehyde in phosphate-buffered saline (PBS: 10 mM sodium phosphate, 0.15 M sodium chloride, pH 7.2). The cells used for Fluorescence Minus One (FMO) condition were stained and acquired in parallel to identify background levels of staining, dead cells were omitted by use of Zombie Aqua^TM^ (BioLegend) viability kit.

The data were collected by means of a FACS Aria II (BD Biosciences, San Jose, CA, USA) or FACS CyAn flow cytometer (Beckman Coulter, Inc. Brea, CA, USA) and analyzed by FlowJo v10.2 (FlowJo LLC, Inc, Ashland, OR, USA). In each case, 50,000 events were acquired per sample. A list of the antibodies clones used can be found in Table S1.

### Cell Sorting

To sort CD3^−^, CD3^+^TCRαβ^−^, and CD3^+^TCRαβ^+^ MDM, single-cell suspensions were obtained from the MDM cultures as described. The cells were incubated in Zombie Aqua^TM^ (BioLegend) for 20 min, at room temperature in the dark. Then, they were stained with BV421-label anti-TCRαβ mAb (clone IP26; BioLegend) and APC-labeled anti-CD3 mAb (clone UCHT1; BioLegend) for 30 min, at 4°C in the dark. Thereafter, the cells in suspension were sorted using a FACS Aria II (BD Biosciences) 85 μm nozzle using the following strategy: The dead cells were excluded by analyzing the negative cell region to the Zombie Aqua staining. The doublets were gated out by plotting the FSC area vs. the FSC height. Finally, the live CD3^−^, CD3^+^ TCRαβ^−^, and CD3^+^TCRαβ^+^ cells were individually sorted.

### Confocal Microscopy

The CD14^+^ cells (1 × 10^6^) were cultured on slides for 7 days at 37°C in a humidified atmosphere containing 5% CO_2_ to obtain MDM, and the cells were washed in PBS and fixed in 2% formaldehyde for 15 min. To avoid unspecific binding, the MDM were blocked for 30 min with 2% porcine serum (Gibco) in PBS (PBS-PS). Posteriorly, the cells were incubated 1 h with primary mAbs to Mannose Receptor (4 μg/mL, Abcam) TCRαβ (4 μg/mL, Thermo Fischer) and CD3 (1:100 cell signaling). The following were used as secondary mAbs (diluted in PBS-PS): donkey anti-rabbit IgG Alexa Fluor-488 (1:100) and donkey anti-rat IgG Alexa Fluor-647 (1:100) provided by Jackson ImmunoReseach and Goat anti-Mouse IgG Alexa Fluor-546 provided by Invitrogen, cells were incubated 1 h. Finally, the samples were washed and incubated with 4_,6-diamidino-2 phenylindole dihydrochloride (DAPI; NucBlue Fixed Cell Stain, Molecular Probes) for 10 min for nuclei labeling and mounting with ProLong Gold Antifade Mountant (Invitrogen). The slides were examined by confocal microscopy FV-1,000 Olympus, and FIJI software was used for the analysis.

### Culture and Stimulation of Sorted Macrophages

Sorted macrophages (1 × 10^5^ cells/200 μl) were incubated for 24 h under different conditions: lipopolysaccharide (LPS) (100 ng/μL) plus interferon-gamma (IFN-γ, 20 ng/mL) (M1 macrophage inducers), anti-CD3 mAb (1 μg/ml) fixed in plate, anti-TNF mAb (1 μg/ml), anti-CD3 mAb plus anti-TNF mAb (1 μg/ml each-one), isotype-matched control mAb (1 μg/ml), or culture medium alone. The culture supernatants were recovered to Bio-Plex cytokine and chemokine analysis; the cells were incubated with an RNA stabilization additive (PAXgene), and posteriorly a qPCR analysis was performed.

### Multiplex Immunoassay

The cell culture supernatants from the human MDM subpopulations that were exposed to the different stimuli, as described above, were recovered and stored at −20°C. A Bio-plex Pro^TM^ Human Cytokine 27-plex assay was carried out following the manufacturer's instructions (Bio-Rad Labs, Hercules, CA, USA). Twenty-seven cytokines and chemokines in culture supernatants were quantified by comparison with appropriated standards. The data were acquired by means of a Bio-Plex 200 System and analyzed using Bio-Plex Manager 6.1 software.

### RNA Extraction and Reverse Transcription

The RNA from macrophages was extracted using an RNeasy Micro Kit (Qiagen, Hilden, Germany) according to the manufacturer's instructions for PAXgene blood RNA. The genomic DNA was eliminated using an RNA-Free DNAse Set (Qiagen). The RNA was eluted in 15 μl of nuclease free water. The quantity of extracted RNA was evaluated by nanodrop. The integrity of the RNA was measured using the Agilent RNA 6000 Pico kit, and the RNA showed RIN greater 5 in each cell subpopulation before and after sorting the cells. Seventy-one nanograms of RNA was used for first-strand cDNA synthesis using the High Capacity cDNA Reverse Transcription Kit (Applied Biosystems, Waltham, USA) in a volume of 20 μl, following manufacturer's guidelines.

### Quantitative Polymerase Chain Reaction

A quantitative real-time PCR was performed with TaqMan probes specifically for the following genes: SOCS3 (SOCS3 gene) (Hs01000485_g1), TCRab (TRBC1 gene) (Hs01588269_g1), and CD3e (CD3e gene) (Hs01062241_m1). ACTB (β-actin) (Hs01060665_g1) and 18S (18S ribosomal RNA gene) (Hs03928990_g1) were used for endogenous control. Single-plex reactions were prepared with Maxima Probe/ROX qPCR Master Mix (Thermo Fisher Scientific, Waltham, USA), and all the amplifications were run in duplicate under the following thermal conditions: 95°C for 10 min followed by 40 cycles of 60°C for 1 min and 95°C for 15 s, with the StepOnePlus^TM^ Real-Time PCR Systems (Applied Biosystems). The relative expression of transcripts was quantified using the ΔCT method, where ΔCT = CT (target) – CT (endogenous). The results were reported as the n-fold change for each target gene in each experimental condition or cell subpopulation, which were normalized to the endogenous controls ACTB and 18S and relative to the control group (= 1).

### Mice

C57BL/6 WT, TNF-deficient (TNF KO) and transmembrane form TNF knock-in (tmTNF KI, TNF^Δ1−9, *K*11*E*^ deletion of amino acids 1–9 and substitution at position 11) mice (10, 14), were maintained under conventional conditions in the animal facility at the Medical Faculty, University of Geneva.

### BCG-Induced Pleurisy

Mycobacterial pleurisy was generated by the intrapleural cavity injection of 10^6^ CFUs of BCG Pasteur in 100 μL of saline solution, as previously reported (9). Injection into the lung parenchyma results in bilateral pneumothorax and death, as mice have only one pleural cavity. The mice were monitored twice a week and sacrificed 2 or 14 weeks after infection. Groups of naïve littermates were killed at the same time and analyzed similarly to the infected mice.

### Pleural Cell and Fluid Preparation

The thoracic cavities from naïve and infected mice were washed with 1 mL 2 mM EDTA–PBS, as previously described (9). The cells were centrifuged, and the pleural cells were suspended in 1% bovine serum albumin-PBS, counted, and prepared to flow cytometry.

### Statistical Methods

The data are shown as median ± standard deviation (SD). A Mann-Whitney U test was used to compare the two groups, and a two-way ANOVA was followed by a Tukey test when more than two groups were compared or with multiple Bonferroni-Dunn comparisons. Values of *p* < 0.05 were considered statistically significant (GraphPad Software, Inc., San Diego, CA, USA).

## Results

### Differentiation of Human Circulating Monocytes in Culture Give Rise to CD3^+^TCRαβ^+^ and CD3^+^TCRαβ^−^ Macrophages

We investigated the frequency of CD3^+^ macrophages generated from human circulating monocytes. Briefly, CD14^+^ cells were enriched (obtaining routinely around 98% of purity), and the absence of CD2^+^ cells was confirmed (Figure S1A). After 7 days in culture, the differentiation process to obtain monocyte-derived macrophages (MDM) was confirmed (Figures S1B,C).

**Figure 1:**
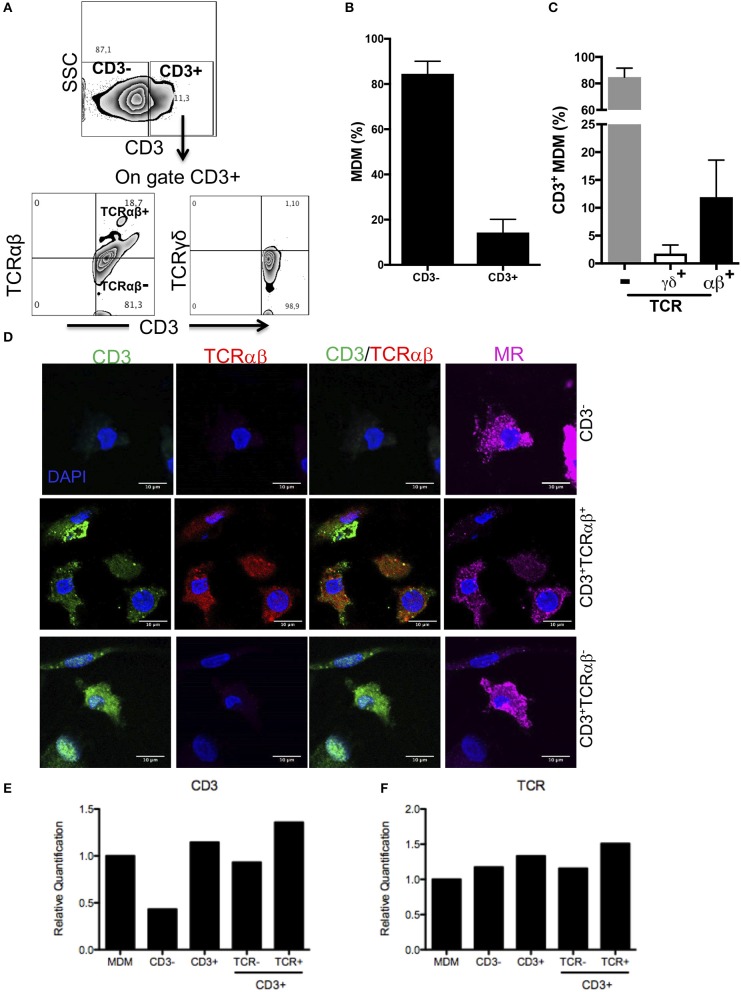
Human MDM give rise to CD3^+^TCRαβ^+^ and CD3^+^TCRαβ^−^ cell subpopulations. Human monocyte-derived-macrophage were obtained, and CD14^+^ cells were harvested after 7 days in culture and prepared for flow cytometry, confocal microscopic or q-PCR. **(A)** Representative zebra plot showing analysis strategy to identify CD3^+^ MDM (up) and co-expression of TCR αβ or γδ chains (down). Frequency of **(B)** CD3^+^ MDM, **(C)** CD3^+^TCRαβ^+^, CD3^+^TCRαβ^−^, and CD3^+^TCRγδ^+^ MDM subpopulations. The bar graphs show the mean ±SD of n = 10 healthy donors. **(D)** Immunofluorescence stained for CD3 (green), TCRαβ (red) and mannose receptor (magenta) and DAPI (blue) was used to identify nucleus. The scale bar 10 μm data are representative of three independent donors. In the purified subpopulation **(E)** CD3 epsilon-chain and **(F)** TCR beta-chain, relative gene expression was evaluated by real-time PCR. The bar graphs show the mean, and the data are representative of two independent donors.

Our analysis strategy to identify CD3 and TCRαβ expression on MDM was as follows: first, we gated CD3^+^ cells, and then the expression of TCRαβ and TCRγδ was evaluated (Figure 1A). Our results showed that ~85% of MDM were CD3^−^ (classic MDM) and only 15% were CD3^+^ cells (Figure 1B); thereafter, we used CD3^−^ MDM as the referenced cell subpopulation for the classic phenotype molecules of MDM. Interestingly, the vast majority of CD3^+^ MDM (~85%) were negative for TCRαβ expression (CD3^+^TCRαβ^−^ MDM), while 12% CD3^+^ MDM were also positive for TCRαβ (CD3^+^TCRαβ^+^ MDM), and <2% expressed TCRγδ (Figure 1C). As the control, TCRαβ and TCRγδ expression was evaluated on gated CD3^−^ MDM, and these cells were negative to TCR expression either αβ or γδ chains (Figure S1D) as expected.

For further confirmation, a co-localization analysis by confocal microscope was developed. The MDM were prepared as described in the Materials and Methods section, and the expression of CD3, TCRαβ and mannose receptor (MR) on MDM was evaluated. The results obtained with this second technique confirmed the presence of CD3^+^TCRαβ^+^ and CD3^+^TCRαβ^−^ MDM subpopulations that also expressed MR (Figure 1D).

As a third technique to confirm the expression of CD3^+^ on macrophages, we evaluated the expression of CD3 and TCRαβ at the transcriptional level. CD3^+^TCRαβ^+^ and CD3^+^TCRαβ^−^ MDM subpopulations were sorted by flow cytometry, RNA was extracted and the integrity was evaluated (RIN >5) (Figure S2). As expected, CD3^−^ MDM had a lower relative expression of CD3 transcript than CD3^+^ MDM, and this was not affected by the absence or presence of TCR at the protein level (Figure 1E). Surprisingly, each MDM subpopulation expressed TCR at the transcriptional level (Figure 1F). Taken together, our analysis confirms that around 15% of the human MDM were CD3^+^ cells, and 85% of them were TCRαβ^−^ cells, and about 12% were TCRαβ^+^ cells.

### CD3^+^TCRαβ^+^ MDM, but Not CD3^+^TCRαβ^−^ MDM, Express Both Non-protein- and Protein-Antigen Presenting Molecules

**Figure 2:**
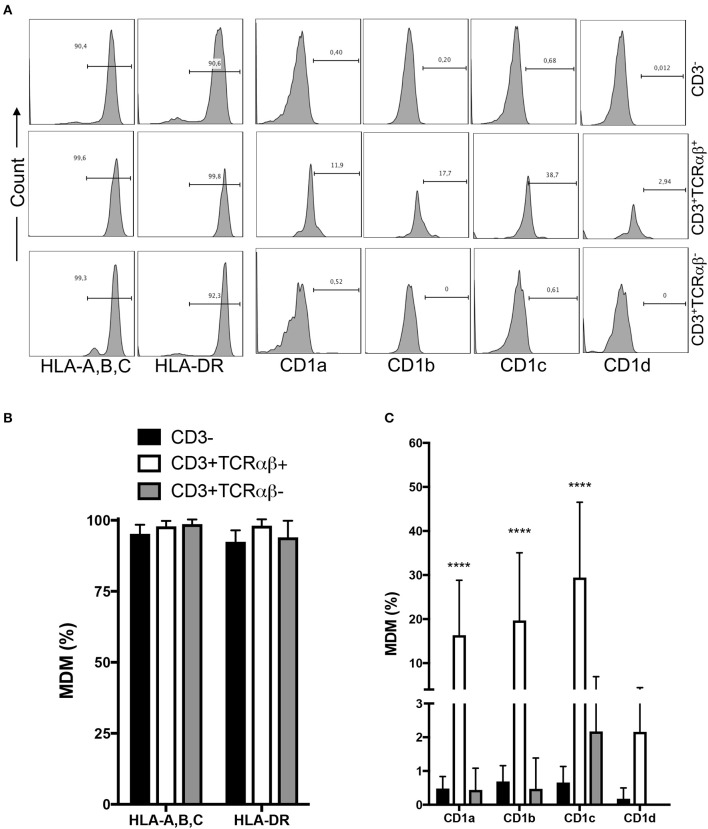
Human CD3^+^TCRαβ^+^ and CD3^+^TCRαβ^−^ macrophage subpopulations express HLA family molecules, but only CD3+TCRαβ+ co-express a CD1 family member. Human monocyte-derived-macrophage were obtained at the 7th day post-culture and prepared for flow cytometry; the CD3^+^ MDM subpopulations were delimited. **(A)** Representative histogram showing the expression of HLA-A,B,C, HLA-DR, CD1a, CD1b, CD1c, and CD1d inside of each CD3^+^ MDM subpopulation. **(B)** Frequency of MDM subpopulations expressing the molecules of the HLA family. **(C)** Frequency of MDM subpopulations expressing the molecules of the CD1 family. The bar graphs show the mean ± SD of *n* = 8–10 independent donors. A statistical analysis was performed by two-way ANOVA with multiple comparisons, followed by a Tukey test. ****p < 0.0001.

Macrophages capture, degrade, and display antigens to activate T cells. Protein antigens are presented by human leukocyte antigen (HLA) class I and II (HLA-A, -B, -C, and HLA-DR, -DP, -DQ, respectively), while non-protein antigens are displayed by members of the CD1 family (CD1a, CD1b, CD1c, and CD1d) molecules (15). To clarify whether MDM subpopulations express HLA and CD1 molecules, we designed gates to identify CD3^+^TCRαβ^−^ and CD3^+^TCRαβ^+^ MDM, and inside each gate, HLA-A,B,C, HLA-DR, CD1a, CD1b, CD1c, and CD1d expression was evaluated by flow cytometry (Figure 2A). As expected, 92–99% of each MDM subpopulation expressed both HLA-A,B,C and HLA-DR (Figure 2B). In contrast, the expression of CD1a, CD1b, and CD1c isoforms, but not CD1d, was exclusive to the CD3^+^TCRαβ^+^ MDM subpopulation (Figure 2C). Our data showed that CD3 expression on MDM did not affect the HLA class I and class II levels on the cell surface. It is noteworthy that the expression of non-protein antigen-presenting molecules was exclusive to CD3^+^TCRαβ^+^ MDM.

### Inflammatory Chemokine Receptors and tmTNF Are Highly Expressed on CD3^+^TCRαβ^+^ MDM

Chemotaxis, a mechanism that drives cell migration to inflammation sites, is essential for immune defense against pathogens and to repair damaged tissues. Beham et al. have previously shown that TCRαβ^+^ macrophages secrete chemokine (C-C motif) ligand 2 (CCL2, or also referred as monocyte chemoattractant protein 1 [MCP1]) by a CD3-dependent pathway to recruit inflammatory monocytes into inflamed tissue (11).

**Figure 3:**
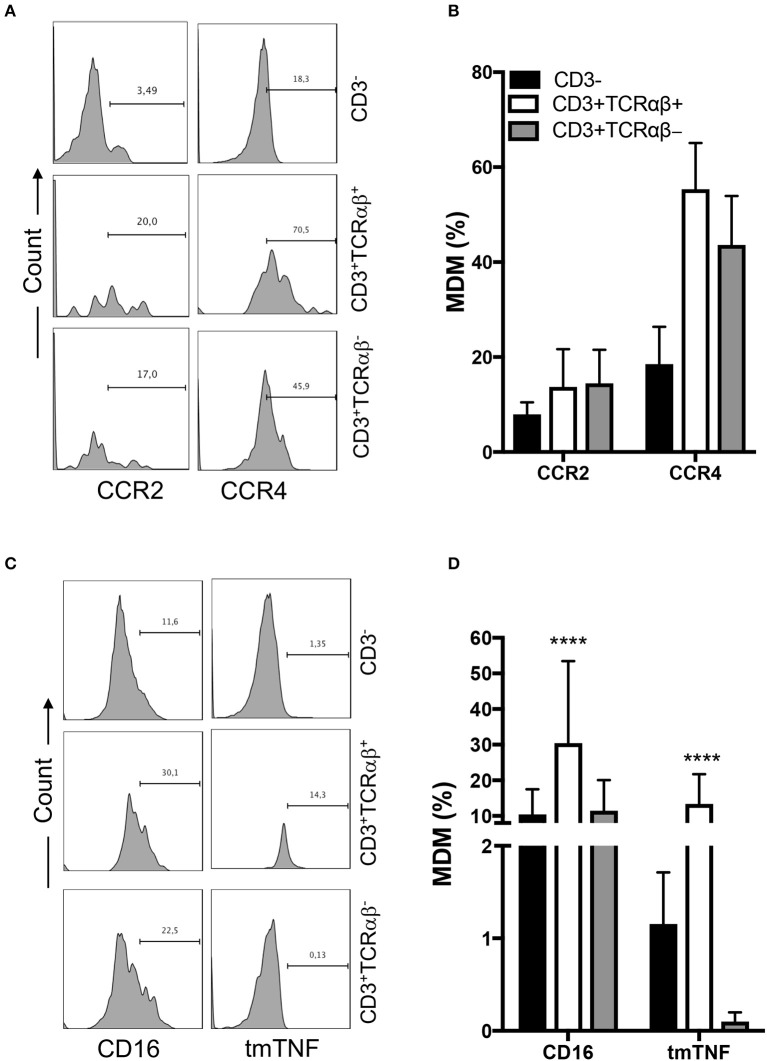
CD3^+^TCRαβ^+^ MDM express chemokine receptors and tmTNF. Human monocyte-derived-macrophage were obtained at the 7th day post-culture and prepared for flow cytometry; the CD3^+^ MDM subpopulations were delimited. **(A)** Representative histogram showing the expression of CCR2 and CCR4 inside of each CD3^+^ MDM subpopulation. **(B)** Frequency of MDM subpopulations expressing chemokine receptors. **(C)** Representative histogram showing the expression of CD16 and tmTNF inside of each CD3^+^ MDM subpopulation. **(D)** Frequency of MDM subpopulation expressing CD16 and tmTNF. The bar graphs show the mean ± SD of *n* = 8–10 independent donors. A statistical analysis was performed by two-way ANOVA with multiple comparisons, following by a Tukey test. ****p < 0.0001.

To assess whether MDM express chemokine receptors, we measured two chemokine receptors by flow cytometry: CC chemokine receptor type 2 (CCR2) and type 4 (CCR4) (Figure 3A). We were not able to identify differences in the frequency of CCR2^+^ and CCR4^+^ MDM, independently of CD3^+^ expression, even when the frequency of CD3^+^ MDM was near double that of CD3^−^ cells; however, the data are not statistically different (Figure 3B).

The expression of CD16 on monocytes has been related to a pro-inflammatory status (16). Thus, we evaluated CD16 and tmTNF expression in each gated cell subpopulation by flow cytometry (Figure 3C). Our data showed that 30% of CD3^+^TCRαβ^+^ MDM expressed CD16, while the CD3^+^TCRαβ^−^ MDM subpopulation had a 10% CD16-expression frequency similar to the CD3^−^ MDM subpopulation (Figure 3D). Also, we found that 10% of CD3^+^TCRαβ^+^ MDM were positive for tmTNF, while tmTNF expression on CD3^+^TCRαβ^−^ MDM was near null, and only 1% of CD3^−^ MDM were tmTNF^+^ (Figure 3D). Together, these results showed that CD3^+^TCRαβ^+^ MDM expressed inflammatory chemokine receptors and also CD16 and tmTNF.

### tmTNF Is Necessary to Recruit CD3^+^ Myeloid Cells at the Infection Site After Pleural BCG Infection

Previously, our group and other have reported that TNF is required to generate TCRαβ^+^ macrophages after *in vitro* BCG infection, and apparently TNFR1 expression is necessary to maintain the presence of CD3^+^ myeloid cell (11, 12). Additionally, our data have shown that CD3^+^TCRαβ^+^ MDM expressed tmTNF (Figure 3D). In this sense, we investigated whether either solTNF or tmTNF was required to maintain the CD3^+^ myeloid cell subpopulations and whether these cell subpopulations can be found specifically at the infection site. Previously, we reported that tmTNF was able to control BCG-induced pleural infections in mice expressing a TNF mutated form (tmTNF KI) that cannot be cleaved by TACE and that does not produce solTNF (10).

**Figure 4:**
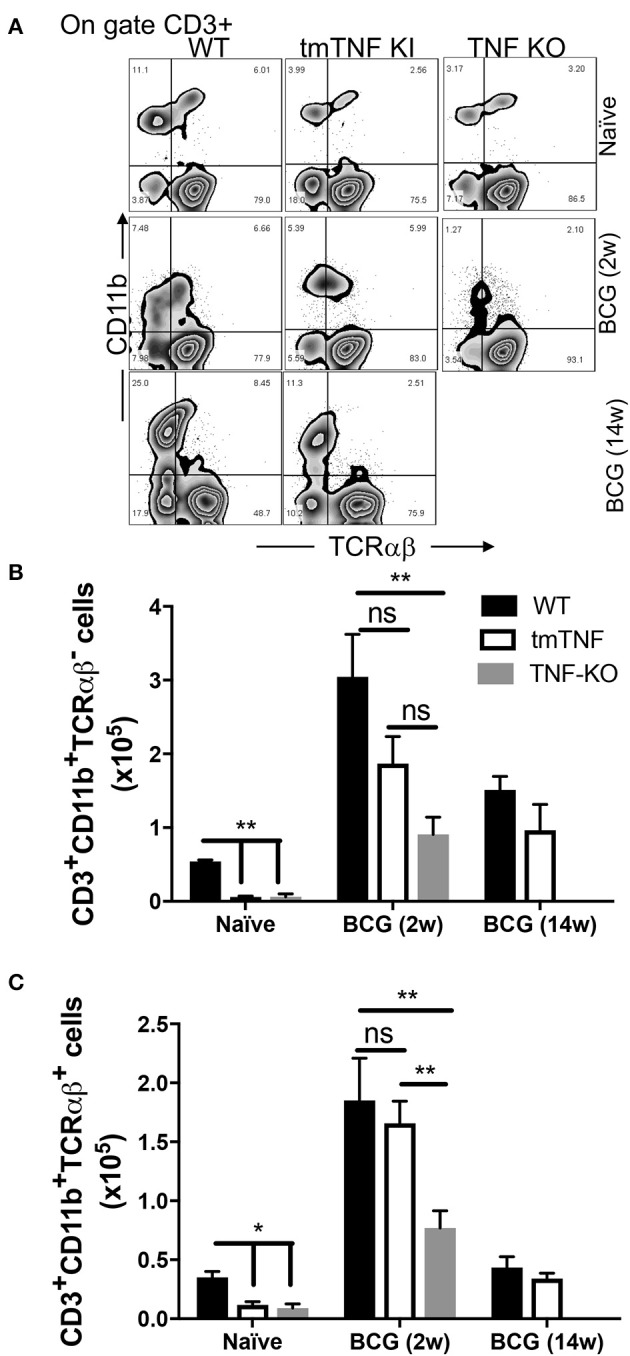
tmTNF regulates the presence of CD3^+^TCRαβ^−^ and CD3^+^TCRαβ^+^ myeloid cells in the pleural cavity after a BCG infection. Wild type (WT), transmembrane TNF (tmTNF KI)-expressing and TNF knockout (TNF KO) mice were infected inside the pleural cavity with BCG. The animals were sacrificed at 2 weeks (BCG 2w) and 14 weeks (BCG 14w) post-infection, and a non-infected group (naïve) was used as the control. **(A)** Representative zebra plot: the CD3^+^ cells were delimited, and the co-expression of CD11b and TCRαβ were evaluated inside this gate in each group. The absolute numbers of CD3^+^CD11b^+^TCRαβ^−^ **(B)** and CD3^+^CD11b^+^TCRαβ^+^ **(C)** subpopulations are shown; these numbers are obtained by measuring the relation between the percentage of cells (obtained by flow cytometry) with the total number of cells recovered from the pleural cavity. The data are expressed as the mean ± SD of *n* = 4–8 mice, three independent experiments. A statistical analysis was performed by two-way ANOVA with multiple comparisons, following by a Tukey test. **p < 0.01; ns = not statistic difference.

The mouse model of BCG-pleural infection was performed, then the pleural cavity cells of WT (wild type), tmTNF KI and TNF KO mice were recovered before and after infection, and the frequency of both CD3^+^CD11b^+^TCRαβ^+^ and CD3^+^CD11b^+^TCRαβ^−^ myeloid cells was assessed by flow cytometry (Figure 4A). We observed that in naïve mice the frequency of both CD3^+^CD11b^+^TCRαβ^+^ and CD3^+^CD11b^+^TCRαβ^−^ myeloid cells was lower in mutant mice compared to WT mice (Figures 4B,C). After infection, WT mice increased 4.5-fold the frequency of CD3^+^TCRαβ^+^ (mean naïve 0.4 × 10^5^ vs. mean 2w 1.8 × 10^5^), and 6-fold the CD3^+^TCRαβ^−^ (mean naïve 0.5X10^5^ vs. mean 2w 3 × 10^5^) myeloid cells at 2 weeks, while at 14 weeks after BCG-infection, the frequency both CD3^+^TCRαβ^+^ and CD3^+^TCRαβ^−^ (0.5 × 10^5^ and 1.2 × 10^5^, respectively) was similar to naïve condition. These results confirm the main role of tmTNF and suggest that CD3^+^ myeloid cells play an important role during the acute phase of BCG infection (Figures 4B,C).

Interestingly, at 2 weeks post-infection the tmTNF KI mice were able to increase the frequency of both MDM subpopulations at the infection site. CD3^+^TCRαβ^−^ myeloid cells were partially recovered in the presence of tmTNF; albeit tmTNF KI mice did not show statistic differences in comparison to TNF KO, also we did not obtained difference between tmTNF KI and WT mice (Figure 4B). In contrast, there is statistical difference when WT is compared with TNF KO (*p* < 0.01), suggesting that tmTNF is enough for a partial recuperating of CD3^+^TCRαβ^−^ myeloid cells during a BCG infection (Figure 4B). Remarkably, the frequency of CD3^+^TCRαβ^+^ myeloid cells was similar in tmTNF KI vs. WT mice and TNF KO, implying that tmTNF is enough to recover the ability to maintain CD3^+^TCRαβ^+^ myeloid cells at the infection site (Figure 4C).

It is important to note that TNF KO mice died at 7–8 weeks post-infection, so we were unable to evaluate the frequency of CD3^+^ myeloid subpopulations at 14 weeks post-infection in TNF KO. However, the frequency of both subpopulations was similar between tmTNF KI and WT mice at 14 weeks post-infection (Figures 4B,C). With this animal model, our data suggested that tmTNF, but not solTNF, is enough for maintaining both CD3^+^ myeloid cell types inside the infection site.

### TNFR2 Expression Increases on CD3^+^CD11b^+^TCRαβ^−^ and CD3^+^CD11b^+^TCRαβ^+^ Myeloid Cells After Pleural BCG Infection

Previously, it has been reported that both TNFR1 and TNFR2 induce cellular activation; however, each receptor apparently activates different signaling pathways. For example, it has been shown that TNFR1 favors pro-inflammatory and pro-apoptotic effects, whereas TNFR2 is involved in regulating cellular activation and proliferation (17, 18). Even under the same context of mycobacterial infection, it has also been demonstrated that they play different roles (10, 19, 20).

Since our results showed that tmTNF is necessary to maintain CD3^+^ myeloid cells in infection site; thus, we wanted to clarify whether the expression of TNFR1 and TNFR2 on the surface of CD3^+^CD11b^+^TCRαβ^+^ and CD3^+^CD11b^+^TCRαβ^−^ myeloid cells is modified between those subpopulations or in response to BCG infection. Therefore, TNFR1 and TNFR2 expression were assessed by flow cytometry in pleural cavity cells of WT, tmTNF KI, and TNF KO mice. First, CD3^+^CD11b^+^TCRαβ^+^ and CD3^+^CD11b^+^TCRαβ^−^ myeloid cells were gated (Figure S3A), posteriorly, inside each of the CD3^+^ myeloid subpopulations the expression of membrane-bound TNFR1 and TNFR2 was assessed in both naïve and BCG-infected mice (Figure S3B).

**Figure 5:**
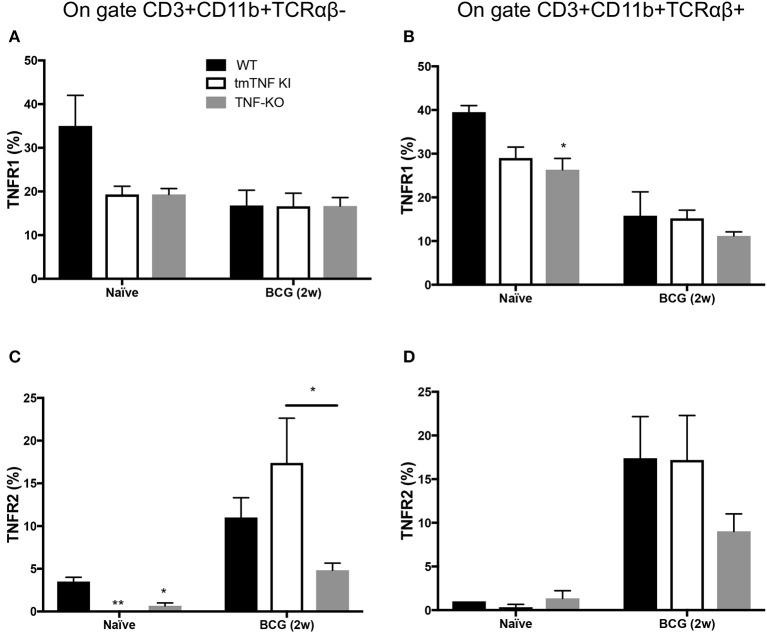
TNFR2 expression increases on CD3^+^TCRαβ^−^ and CD3^+^TCRαβ^+^ myeloid cells in the pleural cavity after pleural BCG infection. Wild type (WT), transmembrane TNF (tmTNF KI)-expressing and TNF knockout (TNF KO) mice were infected inside the pleural cavity with BCG. Animals were sacrificed at 2 weeks (BCG 2w) post-infection, and a non-infected group (naïve) was used as the control. The frequencies of CD3^+^CD11b^+^TCRαβ^−^ and CD3^+^CD11b^+^TCRαβ^+^ positive to TNFR1 (**A,B**, respectively), and the frequencies of CD3^+^CD11b^+^TCRαβ^−^ and CD3^+^CD11b^+^TCRαβ^+^ positive to TNFR2 were reported (**C,D**, respectively). The data are expressed as the mean ± SD of *n* = 4–8 mice, were from three independent experiments. A statistical analysis was performed by two-way ANOVA with multiple comparisons, followed by a Tukey test. *p < 0.05; **p < 0.01.

In naïve mice, both CD3^+^CD11b^+^TCRαβ^−^ and CD3^+^CD11b^+^TCRαβ^+^ cells expressed TNFR1 but mutant mice appeared with a lower frequency, however, only TNF KO showed statistically difference in CD3^+^CD11b^+^TCRαβ^+^ cells in comparison with WT mice (Figures 5A,B). Interestingly, after 2 weeks of BCG-infection, both CD3^+^CD11b^+^TCRαβ^−^ and CD3^+^CD11b^+^TCRαβ^+^ cells decreased TNFR1 expression by ~50% (Figures 5A,B).

TNFR2 expression on CD3^+^CD11b^+^TCRαβ^−^ cells in WT naïve was around 5% and almost null in tmTNF-KI and TNK KO mice (Figure 5C) while CD3^+^CD11b^+^TCRαβ^+^ cells were similar in the three different genotypes (Figure 5D). However, at 2 weeks post infection the TNFR2 expression significantly increased on CD3^+^CD11b^+^TCRαβ^−^ and CD3^+^CD11b^+^TCRαβ^+^ cells. It is noteworthy that the frequency of CD3^+^CD11b^+^TCRαβ^−^ cells positive to TNFR2 was lower in TNF KO mice than in WT mice (Figure 5C).

Together our data show that TNFRs are expressed by both the CD3^+^CD11b^+^TCRαβ^−^ and CD3^+^CD11b^+^TCRαβ^+^ cells, but in response to BCG infection there is a switch from a TNFR1 predominant expression to TNFR2.

### CD3^+^TCRαβ^+^ MDM Produce IL-1β, IP-10, and MCP-1 by CD3- and TNF-Dependent Pathways

Our result showed that human CD3^+^TCRαβ^+^ MDM express tmTNF, and we also showed that tmTNF expression is enough to maintain CD3^+^ myeloid cells during BCG infection using the murine model. We hypothesized that both tmTNF and CD3 molecules on the cell surface probably have the ability to induce the activation of MDM.

**Figure 6:**
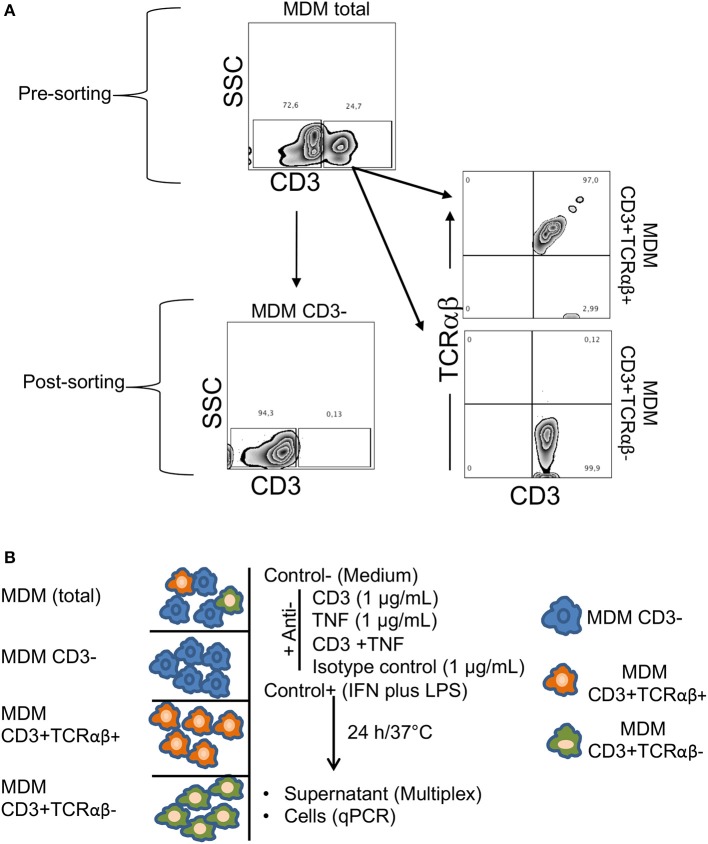
Sorting of CD3^+^TCRαβ^−^ and CD3^+^TCRαβ^+^ MDM using flow cytometry and an experimental strategy. Human MDM were obtained after 7 days in culture, and the cells were prepared to sort CD3^+^TCRαβ^+^, CD3^+^TCRαβ^−^ and CD3^−^ MDM by flow cytometry. **(A)** Representative zebra plot corresponding to total MDM before (up) and after (down, left, and right) sorting. Using adequate controls, the gates were delimited to obtain MDM subpopulations, and our data showed that after sorting, we obtained at least 90% of the enriched cell subpopulation. The data are representative of six independent donors. **(B)** Our experimental design consisted of total and purified MDM cultured and stimulated by anti-CD3 (1 μg/mL), anti-TNF (1 μg/mL), anti-CD3 plus anti-TNF (1 μg/mL each-one) antibodies, isotype control (IgG1) antibody, negative control without stimuli (medium) and a positive control of cell activation (LPS 100 ng/mL plus IFN-γ 20 ng/mL), 24 h in culture under humid conditions (37°C and 5%CO_2_). The supernatant was recovered to measure cytokines and chemokines with Luminex technology, and the cells were recovered to evaluate the gen level expression of the suppressor of cytokine signaling 3 (SOCS3) by q-PCR.

To test this hypothesis, we first obtained MDM from healthy donors, and then we sorted CD3^+^TCRαβ^+^, CD3^+^TCRαβ^−^ and CD3^−^ MDM by flow cytometry. We consistently obtained about 90–99% of purity of the intended cell type (Figure 6A). As a second step, each MDM subpopulation was cultured, and an extra condition with total MDM (before sorting) was included to evaluate the effect of the stimuli when all subpopulations were together. Each MDM subpopulation was stimulated with individual antibodies of anti-CD3 and anti-TNF or anti-CD3 and anti-TNF antibodies together (CD3/TNF). Cytokine and chemokine levels were evaluated in the culture supernatant, and the cells were recovered to measure the gene level expression of the suppressor of the cytokine signaling 3 (SOCS3) molecule, a pro-inflammatory activation marker in macrophages (Figure 6B) (21).

**Figure 7:**
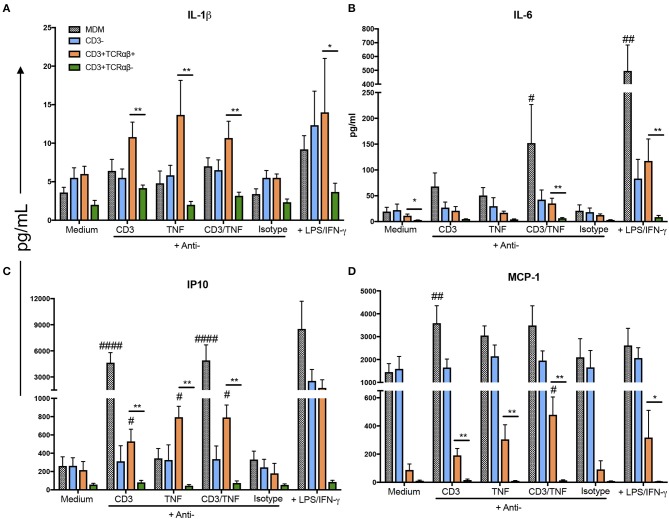
CD3^+^TCRαβ^+^ MDM secrete IL-1β, IP-10, and MCP-1 by CD3- and TNF-dependent pathways. The supernatant of MDM subpopulations was recovered after 24 h in culture from unstimulated or stimulated by anti-CD3 or anti-TNF antibodies or anti-CD3 plus anti-TNF, isotype control and LPS plus IFN-γ, to perform a multiplex analysis. Pro-inflammatory cytokines IL-1β and IL-6 (**A,B**, respectively) and pro-inflammatory chemokines IP-10 and MCP-1 (**C,D**, respectively) were evaluated using Luminex technology. The data are expressed as the mean ± SD of *n* = 4–6 independent donors per condition. A statistical analysis was performed by multiple Bonferroni-Dunn comparisons. *p < 0.05, ^#^*p* < 0.05, **p < 0.01, ^##^*p* < 0.01 and ^####^*p* < 0.0001 (*Indicates difference between groups, ^#^ indicates the difference compared with its negative control condition).

We observed that CD3^+^TCRαβ^+^ MDM secreted higher levels of IL-1β, IL-6, IP-10, and MCP-1 compared to CD3^+^TCRαβ^−^ MDM using anti-CD3 and anti-TNF stimuli (Figure 7). However, each cytokine had its own profile in response to anti-CD3 and anti-TNF antibodies: A high IL-1β level was observed only when the stimulus was on purified CD3^+^TCRαβ^+^ MDM. In contrast, in total MDM, the IL-1β level did not increase (Figure 7A). IL-6 only increased when the cells received anti-CD3 plus anti-TNF stimuli together, and this high level was observed in purified CD3^+^TCRαβ^+^ MDM and total MDM (Figure 7B). IP10 and MCP-1 increased with individual stimulus and the combined stimuli of anti-CD3 and anti-TNF, and those high levels were maintained both in purified CD3^+^TCRαβ^+^ and total MDM (Figures 7C,D). Interestingly, we observed that even when LPS and IFN-γ (positive control) were added to cell stimulation, the CD3^+^TCRαβ^−^ MDM were unable to produce these cytokines and chemokines. Together, our data suggest that CD3^+^TCRαβ^+^ MDM deliver pro-inflammatory cytokines by CD3- and TNF- dependent pathways.

### CD3^+^TCRαβ^−^ MDM Produce IFN-γ, TNF, and MIP-1β by a CD3-Dependent Pathway

Interestingly, CD3^+^TCRαβ^−^ MDM also produced an inflammatory cytokine profile, but it is only by CD3-dependent and TNF-independent pathways. However, CD3^+^TCRαβ^+^ and CD3^+^TCRαβ^−^ MDM deliver their specific profiles of cytokines and chemokines and those profiles do not overlap.

**Figure 8:**
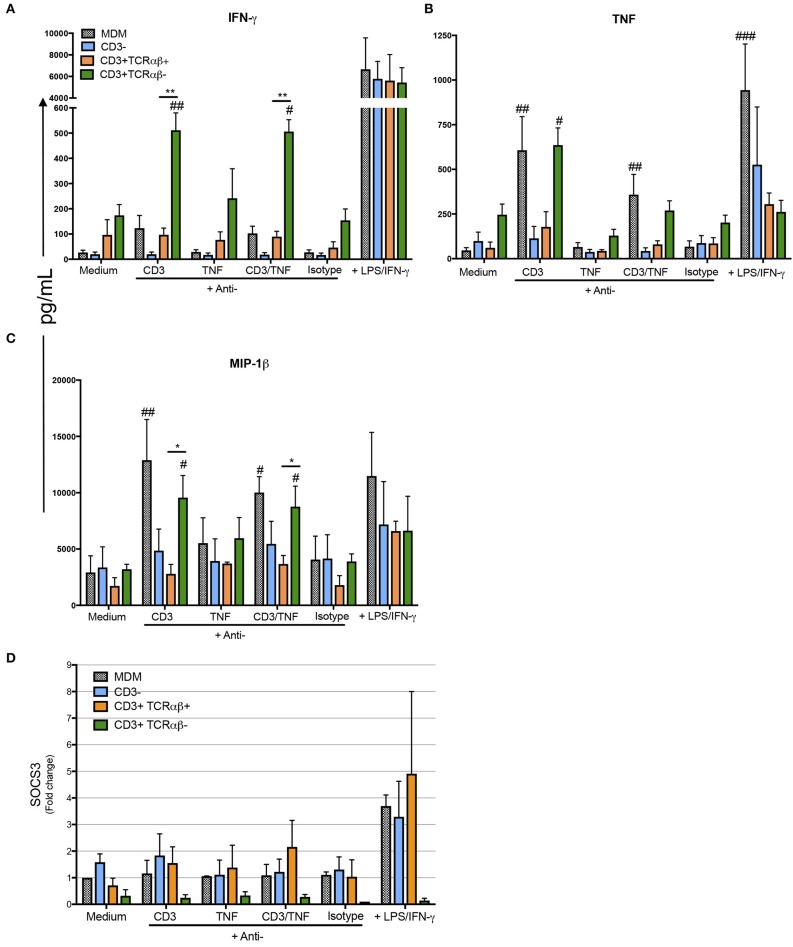
CD3^+^TCRαβ^−^ MDM secrete IFN-γ, TNF, and MIP-1b by CD3- and TNF-dependent pathways. The supernatant of MDM subpopulations was recovered after 24 h in culture from unstimulated or stimulated by anti-CD3 or anti-TNF antibodies or anti-CD3 plus anti-TNF, isotype control and LPS plus IFN-γ, to perform a multiplex analysis, while the cells were prepared to obtain mRNA. Pro-inflammatory cytokines IFN-γ and TNF (**A,B**, respectively) and pro-inflammatory chemokine MIP-1β **(C)** were evaluated using Luminex technology. The data are expressed as the mean ± SD of *n* = 4–6 independent donors per condition. A statistical analysis was performed with multiple Bonferroni-Dunn comparisons. *p < 0.05, ^#^*p* < 0.05, **p < 0.01, ^##^*p* < 0.01, and ^###^*p* < 0.001 (*Indicates the difference between groups, and ^#^indicates the difference compared with its negative control condition). **(D)** The relative gene expression of SOCS3 was evaluated by real-time PCR. The bar graphs show the mean, and the data are representative of three independent donors.

Our data show CD3^+^TCRαβ^−^ MDM specifically produced IFN-γ, TNF, and MIP-1β by a CD3-dependent pathway (Figure 8). However, the high level of IFN-γ was secreted only by purified CD3^+^TCRαβ^−^ MDM and not by total MDM (Figure 8A). On the contrary, TNF and MIP-1β were delivered by purified CD3^+^TCRαβ^−^ and total MDM by means of a CD3-dependent pathway (Figures 8B,C). The anti-inflammatory cytokine levels were measured (IL-1-RA, IL-4, and IL-10), but in our model, we were not able to identify whether CD3^+^TCRαβ^+^ or CD3^+^TCRαβ^−^ MDM secreted an anti-inflammatory profile by CD3- or TNF-dependent pathways (**Figures S4A–C**). Our data suggest both CD3^+^TCRαβ^+^ and CD3^+^TCRαβ^−^ MDM deliver preferably a pro-inflammatory cytokine profile by a CD3-dependent pathway; however, each subpopulation has a specific profile.

For a further confirmation about the pro-inflammatory profile of CD3^+^ MDM subpopulations as a response to anti-CD3 and anti-TNF stimulus, we measured the gene expression level of SOCS3, a marker of pro-inflammatory status in macrophages. Our data indicated that CD3^+^TCRαβ^+^ MDM showed 2–3 fold higher SOCS3 gene expression than CD3^+^TCRαβ^−^ MDM (Figure 8D). We did not obtain statistic differences but it is important to notice that SOCS3 gene expression in CD3^+^TCRαβ^−^ MDM is not activated by any stimulus. On the contrary, CD3^+^TCRαβ^+^ MDM showed a range of gene expression after the stimulation. Interestingly, in total MDM did not increase SOCS3 gene expression with anti-CD3 and anti-TNF but only increased under the LPS/IFN-γ activation. In contrast, CD3^+^TCRαβ^+^ MDM increased two-fold under any stimulus compared to the condition without stimulus (Figure 8D). In summary, CD3^+^TCRαβ^+^ and CD3^+^TCRαβ^−^ MDM secrete proinflamatory cytokines, CD3^+^TCRαβ^+^ MDM showed high dispersion of SOCS3 gene levels in comparison to CD3^+^TCRαβ^−^ MDM.

## Discussion

Although the current knowledge of macrophages is enormous, several questions are still open concerning the nature, function and differentiation process of the diverse macrophage subpopulations (22). The heterogeneity and plasticity of macrophages permit them to adapt and respond to pro- and anti-inflammatory microenvironments, and they are related to diverse pathologies according to the macrophage profile (23).

Macrophages express on the cell surfaces a wide array of receptors that help to define their function. In 2006, Puellmann et al. reported for the first time the expression of TCR on the cell surface of human neutrophils, suggesting the controversial existence of myeloid cells expressing molecules that traditionally have been related to cells from the adaptive immune system (24). Later, Beham et al. showed that a small subpopulation of human circulating monocytes and macrophages can also express an unusual TCRαβ generated by V(D)J recombination, and provided evidence for the first time of its implication in such major infectious diseases as TB (11). At present, a growing body of evidence supports the presence of TCR^+^ myeloid cells in healthy or pathological conditions, as discussed below (24–28).

Here, we demonstrated that a small fraction of circulating monocytes from a healthy donor is typically differentiated into CD3^+^ MDM, and this cell subpopulation can be divided into TCRαβ^+^ and TCRαβ^−^ phenotypes, both subpopulations has been recently reported in mice and they are negative to CD2 (12). In this regard, we found both proteins and mRNA transcripts for TCR chains in each MDM subpopulation, suggesting that MDM have the ability to express TCR on macrophages under an appropriate stimulus. In addition, a recent publication has reported that the intensity of expression of CD3 on murine myeloid cells is lower compared to the intensity level on lymphocytes (12). We have to consider that even with an efficient macrophage activation, classic CD3^+^ T cells will express higher levels of CD3 and TCR than macrophages.

Moreover, our data show that both CD3^+^ macrophage subpopulations express HLA class I and II molecules, indicating that they have the ability to present antigens to other cells. Even if we did not evaluate the antigen presentation function, previous reports have shown that CD3^+^ macrophages are capable of this activity (11, 12). Surprisingly, we find that CD3^+^TCRαβ^+^ MDM, but not CD3^+^TCRαβ^−^ MDM, expressed CD1a, CD1b, and CD1c, which are non-protein antigen-presenting molecules, suggesting that macrophages are ready to respond using multiple resources against pathogens, which cannot be eliminated by conventional microbicidal mechanisms. In this way, it is possible that the frequency of CD3^+^ macrophages increases in response to *M. tuberculosis* infection because the cell wall of this pathogen contains abundant lipids. Moreover, it has been wide described that CD1 family molecules are important in the context of mycobacterial infections (29, 30). A recent report has shown that the role of CD3^+^ macrophages is not limited to infectious pathologies, since these cells have been implicated in atherosclerosis. The authors showed that CD3^+^ macrophages accumulated on a carotid artery lesion, and cholesterol played a role in modulating the TCR expression repertoire (31). It is reasonable to think that the role of CD3^+^ macrophages in atherosclerosis is mediated by CD1 family expression, opening a new field to study this cellular subpopulation in pathologies where lipid plays a main role in the physiopathology.

Previously, Beham et al. showed that total macrophages stimulated by anti-CD3 Ab produced CCL2, which is an important chemokine in the attraction of circulating monocytes at the site of infection (11). Here, using a purified MDM subpopulation, we demonstrated that both CD3^+^ MDM (TCRαβ^+^ and TCRαβ^−^) subpopulations are able to produce pro-inflammatory cytokines by CD3- and/or TNF-dependent pathways. However, CD3^+^TCRαβ^+^ MDM specifically produced two relevant chemokines to recruit cells at the infection site, IP10 and MCP-1. Moreover, CD3^+^TCRαβ^−^ MDM delivered IFN-γ, TNF and MIP-1β, which are important to cellular activation. Due to the specific cytokine profile delivered by each subpopulation, we speculate that CD3^+^TCRαβ^+^ and CD3^+^TCRαβ^−^ MDM help to activate the inflammatory process through the recruitment of different cell populations. While MCP-1 is delivered specifically by CD3^+^TCRαβ^+^ MDM, MIP-1β is secreted by CD3^+^TCRαβ^−^ MDM, suggesting that each cell population plays a specific role for migration and cellular activation.

Recently, it has been reported that TCRαβ-expressing macrophages accumulate in the brain during experimental cerebral malaria, but in contrast to our data, those macrophages are mainly TCRαβ^+^, not CD3ε cells. This discrepancy could be due to different anatomic origins (brain vs. pleural cavity) or the nature of the cells. Indeed, we evaluated the total myeloid cells in the mouse (CD11b^+^), while Oakley et al. limited their study specifically to macrophages (CD11b^high^CD14^+^F4/80^+^) (26).

In this study, we demonstrated that after a BCG-induced pleural infection, both CD3^+^ myeloid cell types can be found at the infection site. Thus, our data concur with those of another report that showed the migration ability of these cells, but it is still unclear whether differences exist between the two cell subpopulations, as suggested by the different profiles of chemokine receptors expressed. Additionally, our work clearly demonstrated that only tmTNF, but not soluble TNF, plays a main role to regulate the presence of CD3^+^ myeloid cells at local infection site, nevertheless our model did not clarify the origin of those CD3^+^ myeloid cells, because they can be recruited from circulating cells or they were expanded *in situ* or both. Also, it is important to consider that a switch occurs between TNFR1 and TNFR2 expressed by CD3^+^ myeloid cells. It is possible that the axis tmTNF/TNFRs is also helpful in regulating this cell migration, in concordance with our previous data, it is possible that TNFR1 is important to recruit CD3^+^ myeloid cells at the infection site, and probably TNFR2 is involved to cell activation (10, 12).

At present, it is not clear whether the migration or function of CD3^+^TCRαβ^+^ and CD3^+^TCRαβ^−^ MDM is mediated only by TNFR1, TNFR2 or both together. We have reported that the axis tmTNF/TNFRs can be self-regulated during BCG-induced pleurisy, tmTNF restores the normal expression of TNFR2 on myeloid cells, and in turn, the absence of TNFR1 affects the expression of TNFR2, leading to exacerbated inflammation and bad control of the infection (32). We have also shown that in the context of BCG pleural infection, the interaction of tmTNF-expressing MDSC with CD4^+^ T cells expressing TNFR2 (expression of TNFR1 was dispensable) leads to attenuating the excessive inflammatory response (10). However, it was reported that TNFR1 expression on myeloid cells is the first line of defense against a *M. tuberculosis* infection (20).

Important questions are still open after this study, and we consider of interest to clarify the axis tmTNF/TNFRs to identify the signaling pathways in the context of CD3^+^ myeloid cells that could be helpful for new insights on the role and mechanism of activation and recruitment of these non-classic myeloid cells.

**Figure 9:**
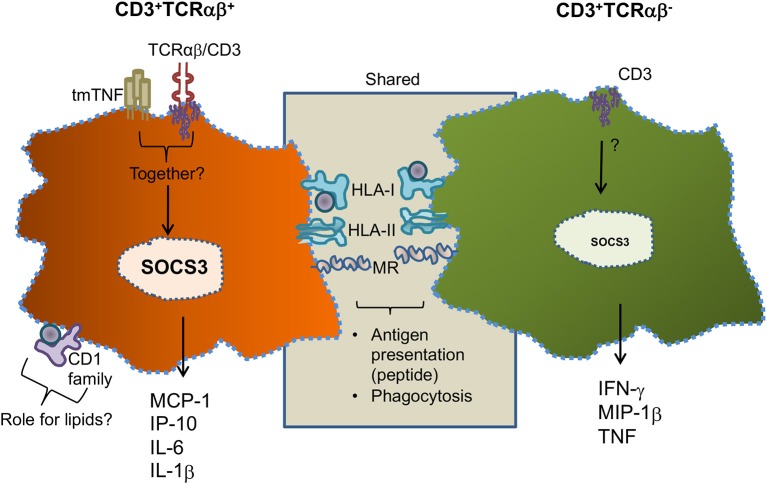
Schematic representation of specific and shared characteristics between CD3^+^TCRαβ^+^ and CD3^+^TCRαβ^−^ MDM. Human circulating monocytes can be differentiated into CD3^+^TCRαβ^+^ and CD3^+^TCRαβ^−^ MDM; both macrophage subpopulations share characteristics, such as the expression of classic-MDM' molecules, protein antigen presenting molecules (HLA family), and mannose receptors (MR). However, CD3^+^TCRαβ^+^ MDM co-express molecules of the CD1 family and secrete MCP1, IP-10, IL-6, and 1L-1β by tmTNF- and CD3-dependent pathways **(left)**. CD3^+^TCRαβ^−^ MDM do not express tmTNF, but specifically secrete IFN-γ, MIP1-β, and TNF by a CD3-dependent pathway **(right)**. At this point, it is unclear whether tmTNF and CD3 act co-dependently to induce the activation of cytokines in CD3^+^TCRαβ^+^ MDM, but the SOCS3 expression is increased.

Figure 9 is a summary of our main data in this work. Human circulating monocytes can give origin to a small subpopulation of MDM-expressing CD3 cells, and this cellular subpopulation can be divided into CD3^+^TCRαβ^+^ and CD3^+^TCRαβ^−^ MDM. Both CD3^+^ MDM subpopulations share the characteristics of classic-MDM (CD3^−^ macrophage), including the expression of molecules such as HLA-I, HLA-II, and MR, indicating their role in phagocytosis and antigen presentation. However, CD3^+^TCRαβ^+^ MDM additionally co-express CD1 family molecules, indicating a role in pathologies where lipids are abundant. CD3^+^TCRαβ^+^ MDM express tmTNF and CD3 which are involved in MDM activation and under activation specifically secrete MCP1, IP-10, IL-6, and IL-1β. In contrast, CD3^+^TCRαβ^−^ MDM do not express tmTNF, but the CD3 expressed on cell surfaces also induces their activation and the secretion of inflammatory cytokines. However, they specifically deliver IFN-γ, MIP1-β, and TNF (Figure 9). It is important to note that the cytokine/chemokine profiles of CD3^+^TCRαβ^+^ and CD3^+^TCRαβ^−^ MDM do not overlap, suggesting that each of these cell subpopulations plays a different role during mycobacterial infections.

## Data Availability Statement

The raw data supporting the conclusions of this manuscript will be made available by the authors, without undue reservation, to any qualified researcher.

## Ethics Statement

This study was approved by the Institutional Review Board (IRB# B07-16) and by Cantonal Veterinary office from Geneva (GE167/14).

## Author Contributions

Conception and drafting of the article: IG and LC-G. Performance and analysis of experiments: AR-C, DV, LR-L, and LC-G. Discussions of the data and critical revision of the article: RL, JZ, VQ, BR, IG, and LC-G. Contribution of reagents, materials, and analysis tools: LR-L, JZ, RL, and IG. Wrote the manuscript: IG and LC-G.

### Conflict of Interest

The authors declare that the research was conducted in the absence of any commercial or financial relationships that could be construed as a potential conflict of interest.

## Supplementary Material

Figure S1 Differentiation process to obtain monocyte-derived-macrophages (MDM). **(A)** CD14^+^ monocytes were enriched by magnetic beads-labeled antibodies; representative histograms show the purity percentage (left) and verification of absence of exclude lymphoid cells in our culture (right). **(B)** After 7 days in culture, MDM morphology was confirmed by microscopy using a magnification 20X (left) and 100X (right, scale bar 10 μm). **(C)** Representative zebra plot and histogram of MDM where the expression of CD68, CD14, HLA-DR, CD11b, CD80, and CD86 were measured by flow cytometry. **(D)** Representative zebra plot to evaluate the expression of TCR αβ and γδ chains inside the CD3^−^ gated cells. The data are representative of at least 12 independent donors.

Figure S2 High quality of RNA to develop qPCR for each MDM subpopulation, before and after FACS sorting. Human MDM subpopulations were sorted by flow cytometry, using the Agilent RNA 6000 Pico kit, and the RNA integrity number (RIN) was obtained using an Agilent bioanalyzer to extract an algorithm that describes RNA integrity. RIN was obtained from human MDM before sorting (total MDM) **(A)**, and after sorting in CD3^+^TCRαβ^−^ MDM **(B)** and CD3^+^TCRαβ^+^ MDM **(C)**. The RIN value is squared in blue. The data are representative of three independent donors.

Figure S3 Analysis strategy to evaluate TNF-receptors on CD3^+^CD11b^+^TCRαβ^−^ and CD3^+^CD11b^+^TCRαβ^+^ cells after a BCG-pleural infection. A BCG-pleural infection was induced, and the cells from the pleural cavity were recovered and prepared for flow cytometry. Representative zebra plot from WT mice after 2 weeks post-infection; with adequate isotype control antibodies, the CD3^+^ cells were gated, and subsequently, the co-expression of CD11b and TCRαβ was identified **(A)**. Representative zebra plot from wild type (WT), transmembrane TNF (tmTNF KI)-expressing, and TNF knock-out (TNF KO) mice. The animals were sacrificed at 2 weeks (BCG 2w). A non-infected group (naïve) was used as the control. Inside the gate to CD3^+^CD11b^+^TCRαβ^−^ and CD3^+^CD11b^+^TCRαβ^+^ MDM, the expression of TNFR1 and TNFR2 was evaluated. The data represent four to eight animals per group from three independent experiments.

Figure S4 CD3^+^TCRαβ^+^ and CD3^+^TCRαβ^−^ MDM do not secrete anti-inflammatory cytokines by CD3- and TNF-dependent pathways. The supernatant and MDM subpopulations were recovered after 24 h in culture stimulated by anti-CD3 and anti-TNF antibodies and prepared to develop a multiplex analysis. Anti-inflammatory cytokines IL-1-RA, IL-4, and IL-10 (**A–C**, respectively) were evaluated using Luminex technology. The data are expressed as the mean ± SD of *n* = 4–6 independent donors per condition.

Table S1 Clones and flurochomes of antibodies.
