# Supplementary figures and images for: CD3+ Macrophages Deliver Proinflammatory Cytokines by a CD3- and Transmembrane TNF-Dependent Pathway and Are Increased at the BCG-Infection Site
Source: Front Immunol. 2019 Nov 7;10:2550. doi: 10.3389/fimmu.2019.02550 (PMC6855269; doi:10.3389/fimmu.2019.02550)

# A CD14+ cells isolated

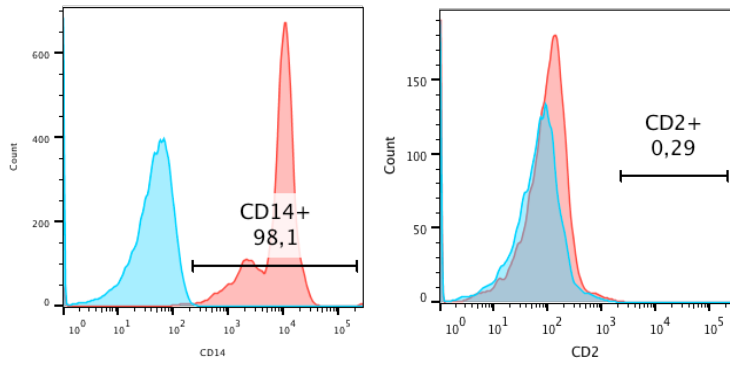

# B 7th Day post-culture

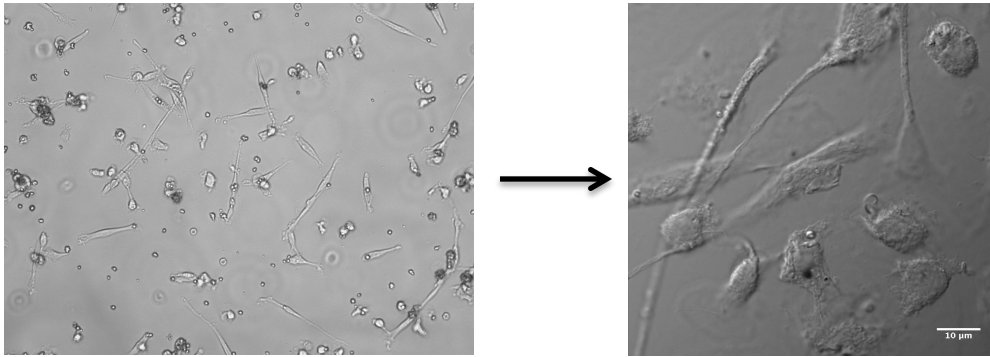

# C

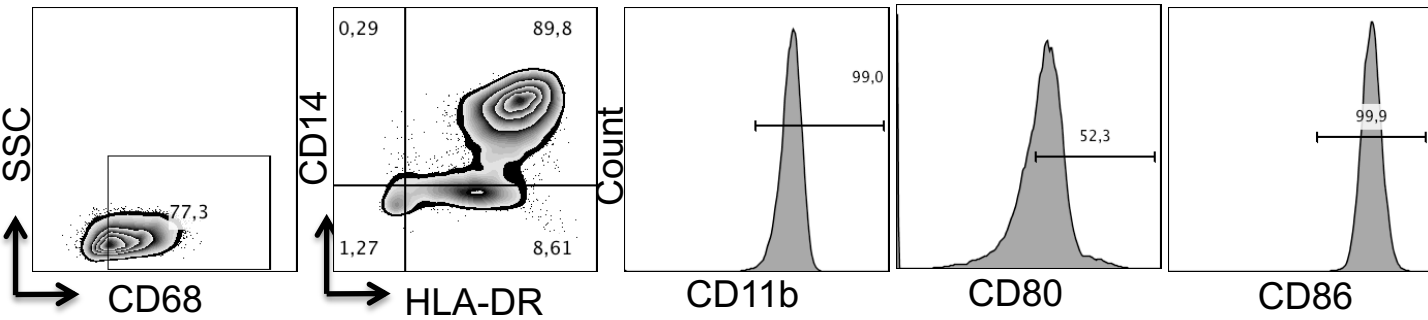

# D

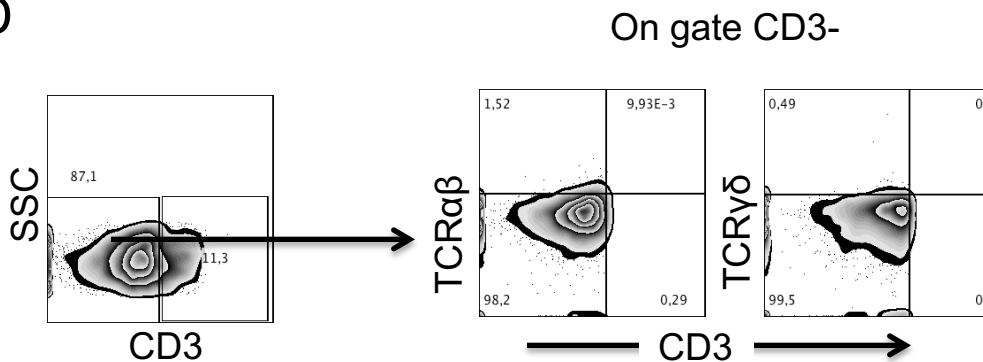

FIGURE S1

Supplement: Figure S1 — Differentiation process to obtain monocyte-derived-macrophages (MDM). (A) CD14+ monocytes were enriched by magnetic beads-labeled antibodies; representative histograms show the purity percentage (left) and verification of absence of exclude lymphoid cells in our culture (right). (B) After 7 days in culture, MDM morphology was confirmed by microscopy using a magnification 20X (left) and 100X (right, scale bar 10 μm). (C) Representative zebra plot and histogram of MDM where the expression of CD68, CD14, HLA-DR, CD11b, CD80, and CD86 were measured by flow cytometry. (D) Representative zebra plot to evaluate the expression of TCR αβ and γδ chains inside the CD3− gated cells. The data are representative of at least 12 independent donors. [file Image_1.pdf]

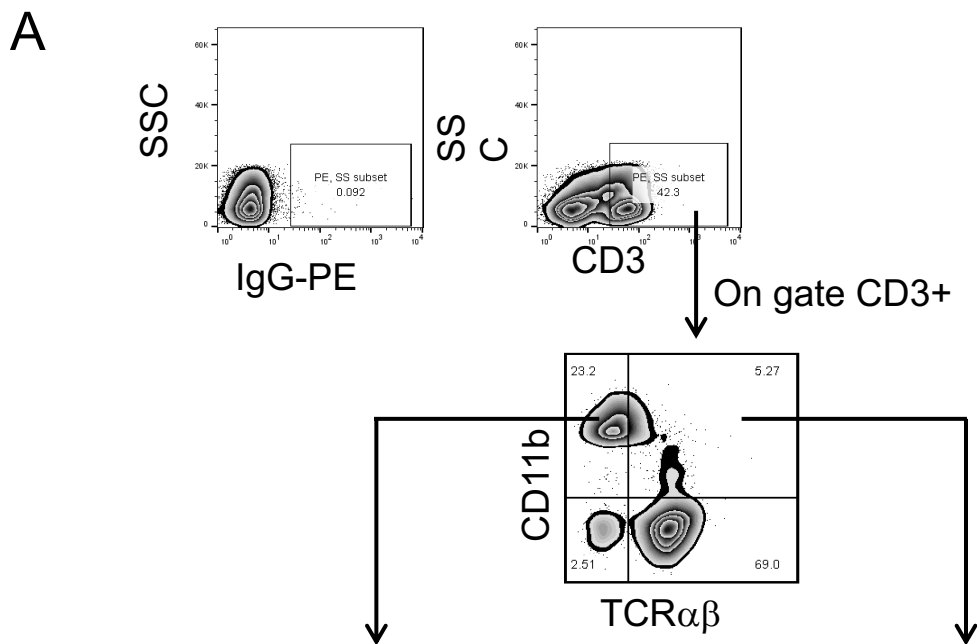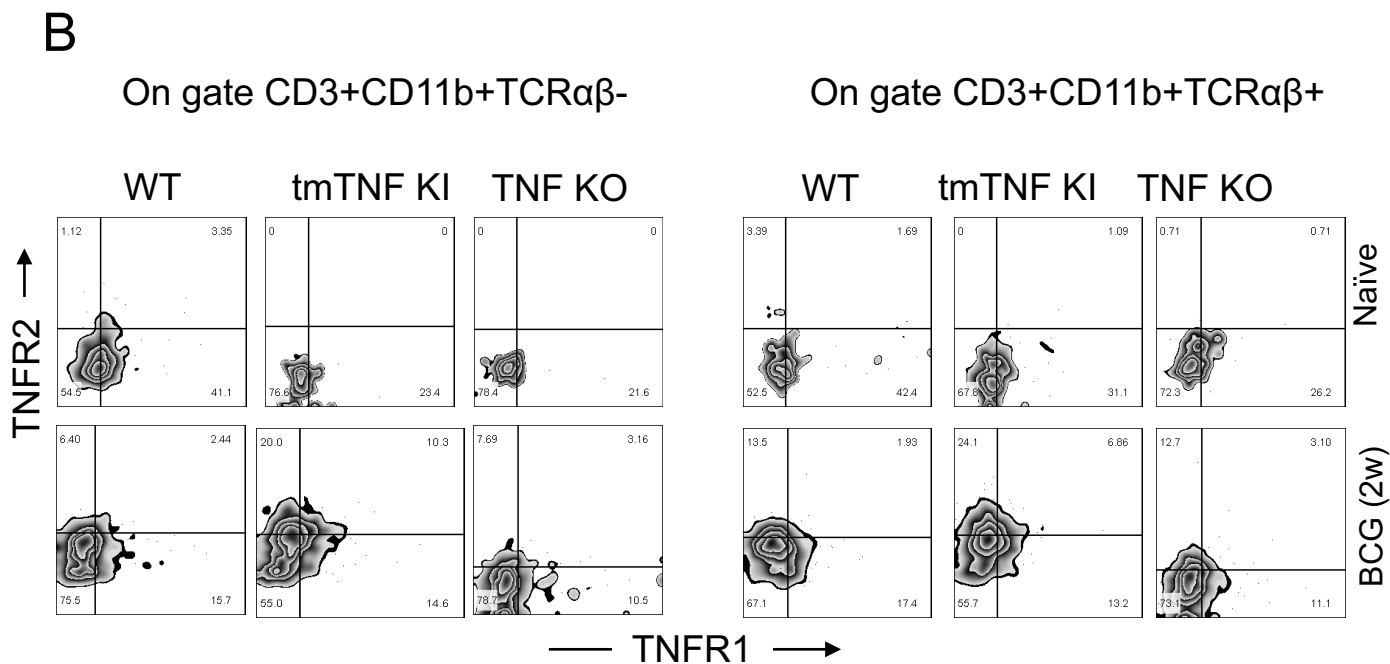

FIGURE S3

Supplement: Figure S3 — Analysis strategy to evaluate TNF-receptors on CD3+CD11b+TCRαβ− and CD3+CD11b+TCRαβ+ cells after a BCG-pleural infection. A BCG-pleural infection was induced, and the cells from the pleural cavity were recovered and prepared for flow cytometry. Representative zebra plot from WT mice after 2 weeks post-infection; with adequate isotype control antibodies, the CD3+ cells were gated, and subsequently, the co-expression of CD11b and TCRαβ was identified (A). Representative zebra plot from wild type (WT), transmembrane TNF (tmTNF KI)-expressing, and TNF knock-out (TNF KO) mice. The animals were sacrificed at 2 weeks (BCG 2w). A non-infected group (naïve) was used as the control. Inside the gate to CD3+CD11b+TCRαβ− and CD3+CD11b+TCRαβ+ MDM, the expression of TNFR1 and TNFR2 was evaluated. The data represent four to eight animals per group from three independent experiments. [file Image_3.pdf]

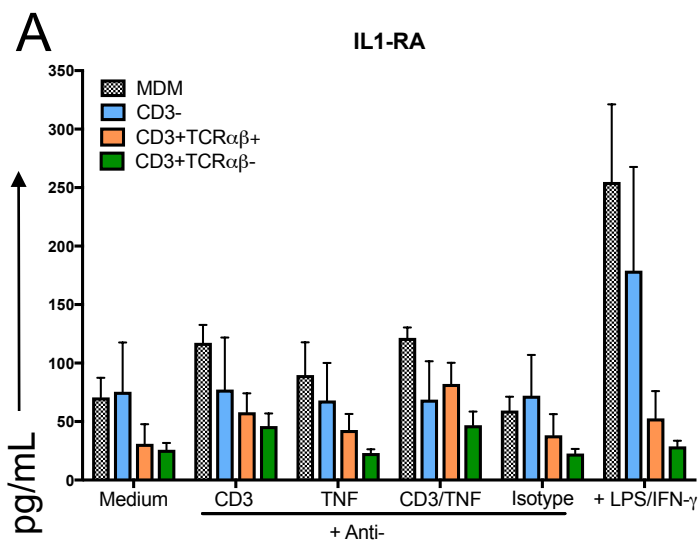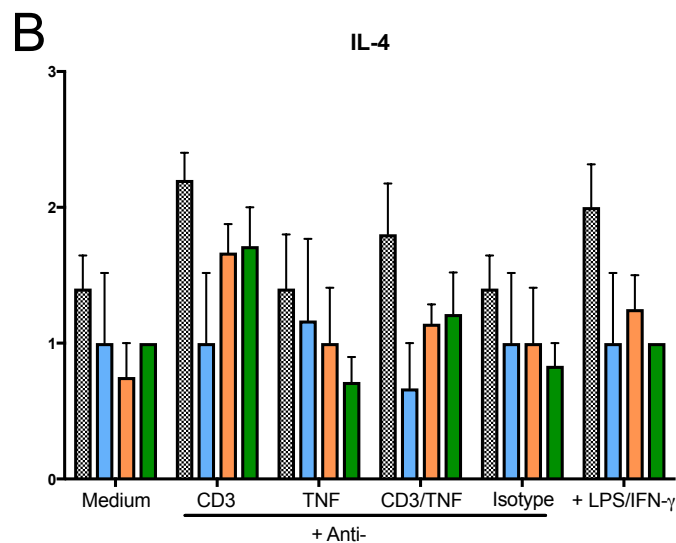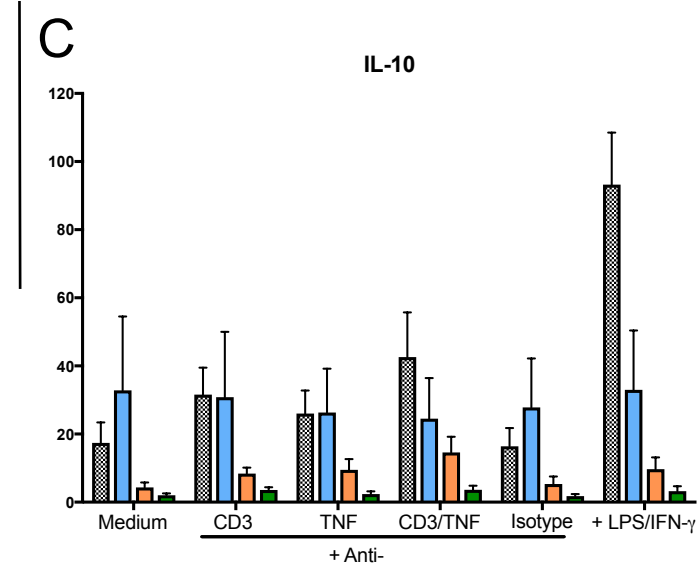

FIGURE S4

Supplement: Figure S4 — CD3+TCRαβ+ and CD3+TCRαβ− MDM do not secrete anti-inflammatory cytokines by CD3- and TNF-dependent pathways. The supernatant and MDM subpopulations were recovered after 24 h in culture stimulated by anti-CD3 and anti-TNF antibodies and prepared to develop a multiplex analysis. Anti-inflammatory cytokines IL-1-RA, IL-4, and IL-10 (A–C, respectively) were evaluated using Luminex technology. The data are expressed as the mean ± SD of n = 4–6 independent donors per condition. [file Image_4.pdf]
