# Supplementary material for: CD3+ Macrophages Deliver Proinflammatory Cytokines by a CD3- and Transmembrane TNF-Dependent Pathway and Are Increased at the BCG-Infection Site
Source: Front Immunol. 2019 Nov 7;10:2550. doi: 10.3389/fimmu.2019.02550 (PMC6855269; doi:10.3389/fimmu.2019.02550)
Supplement: Figure S2 — High quality of RNA to develop qPCR for each MDM subpopulation, before and after FACS sorting. Human MDM subpopulations were sorted by flow cytometry, using the Agilent RNA 6000 Pico kit, and the RNA integrity number (RIN) was obtained using an Agilent bioanalyzer to extract an algorithm that describes RNA integrity. RIN was obtained from human MDM before sorting (total MDM) (A), and after sorting in CD3+TCRαβ− MDM (B) and CD3+TCRαβ+ MDM (C). The RIN value is squared in blue. The data are representative of three independent donors. [file Image_2.pdf]

A

Total MDM (before sorting)

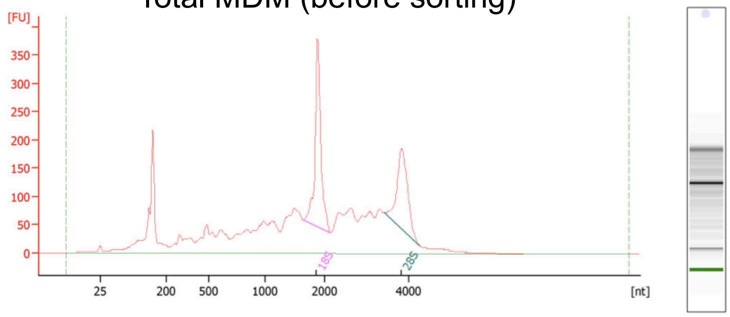


**Overall Results for sample 2 :** Sample 2

| | | | |
|---|---|---|---|
| RNA Area: | 3,999.8 | RNA Integrity Number (RIN): | 6.4 (B.02.08) |
| RNA Concentration: | 18,764 pg/µl | Result Flagging Color: | |
| rRNA Ratio [28s / 18s]: | 0.7 | Result Flagging Label: | RIN: 6.40 |

**Fragment table for sample 2 :** Sample 2

| Name | Start Size [nt] | End Size [nt] | Area | % of total Area |
|---|---|---|---|---|
| 18S | 1,639 | 2,134 | 487.6 | 12.2 |
| 28S | 3,416 | 4,275 | 318.3 | 8.0 |

B

CD3$^{+}$TCR$\alpha\beta^{-}$ MDM (after sorting)

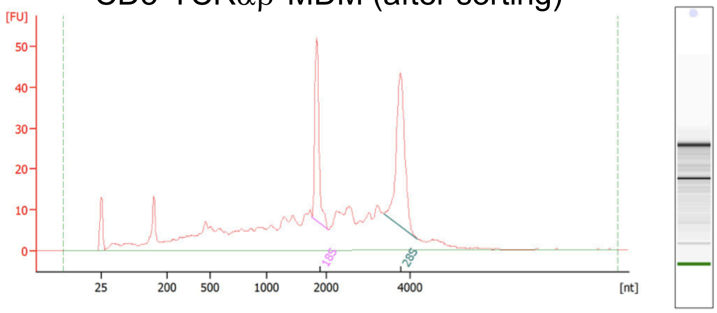


**Overall Results for sample 4 :** Sample 4

| | | | |
|---|---|---|---|
| RNA Area: | 511.0 | RNA Integrity Number (RIN): | 6.6 (B.02.08) |
| RNA Concentration: | 2,397 pg/µl | Result Flagging Color: | |
| rRNA Ratio [28s / 18s]: | 1.5 | Result Flagging Label: | RIN: 6.60 |

**Fragment table for sample 4 :** Sample 4

| Name | Start Size [nt] | End Size [nt] | Area | % of total Area |
|---|---|---|---|---|
| 18S | 1,756 | 2,037 | 44.9 | 8.8 |
| 28S | 3,364 | 4,181 | 67.5 | 13.2 |

C

CD3$^{+}$TCR$\alpha\beta^{+}$ MDM (after sorting)

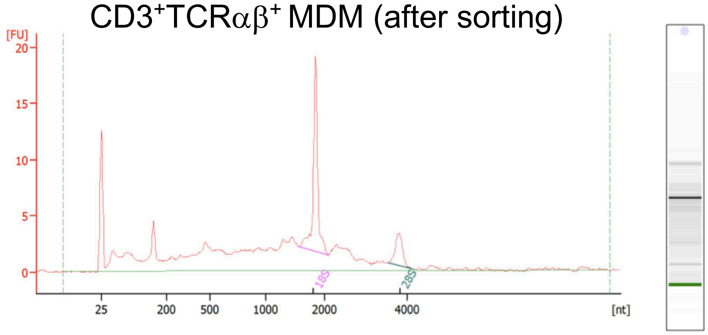


**Overall Results for sample 5 :** Sample 5

| | | | |
|---|---|---|---|
| RNA Area: | 146.8 | RNA Integrity Number (RIN): | 6.3 (B.02.08) |
| RNA Concentration: | 689 pg/µl | Result Flagging Color: | |
| rRNA Ratio [28s / 18s]: | 0.2 | Result Flagging Label: | RIN: 6.30 |

**Fragment table for sample 5 :** Sample 5

| Name | Start Size [nt] | End Size [nt] | Area | % of total Area |
|---|---|---|---|---|
| 18S | 1,565 | 2,080 | 19.5 | 13.3 |
| 28S | 3,521 | 4,134 | 4.5 | 3.1 |

FIGURE S2
