# Supplementary material for: CD3+ Macrophages Deliver Proinflammatory Cytokines by a CD3- and Transmembrane TNF-Dependent Pathway and Are Increased at the BCG-Infection Site
Source: Front Immunol. 2019 Nov 7;10:2550. doi: 10.3389/fimmu.2019.02550 (PMC6855269; doi:10.3389/fimmu.2019.02550)
Supplement: Table S1 — Clones and flurochomes of antibodies. [file Table_1.pdf]

Table 1. Clone and fluorochrome of antibodies

Antigen	Reactivity	Clone	Fluorochrome	Manufacturer
CD80	Human	2D10	APC	Biolegend
CD86	Human	IT2.2	PE Cy7	Biolegend
CD11b	Human	ICRF44	PerCP Cy5.5	Biolegend
CD68	Human	Y1/82A	PE	Biolegend
CD14	Human	63D3	FITC	Biolegend
CD14	Human	M5E2	BV510	Biolegend
CD16	Human	3G8	PE Cy7	Biolegend
HLA-DR	Human	L243	APC	Biolegend
HLA-A,B,C	Human	W6/32	PE Cy7	Biolegend
CD3 ε	Human	HIT3a	APC Cy7	Biolegend
TCRab	Human	IP26	FITC	Biolegend
TCRab	Human	IP26	BV421	Biolegend
TCRgd	Human	B1	PE	Biolegend
CD1a	Human	HI149	PerCP Cy5.5	Biolegend
CD1b	Human	SN13	FITC	Biolegend
CD1c	Human	L161	PerCP Cy5.5	Biolegend
CD1d	Human	51.1	PE	Biolegend
CD2	Human	TS1/8	BV421	Biolegend
CCR2	Human	K036C2	PE	Biolegend
CCR4	Human	L291H4	PE Cy7	Biolegend
TNF	Human	Mab11	BV421	Biolegend
CD11b	Mice	M1/70	FITC	Biolegend
CD3 ε	Mice	145-2C11	APC Cy7	Biolegend
TCRab	Mice	H57-597	PE Cy7	Biolegend
TNFR1	Mice	55R-286	APC	Biolegend
TNFR2	Mice	TR75-89	PE	Biolegend
